# Comparison of dosimetric impact of intra‐fractional setup discrepancy between multiple‐ and single‐isocenter approaches in linac‐based stereotactic radiotherapy of multiple brain metastases

**DOI:** 10.1002/acm2.13484

**Published:** 2021-12-20

**Authors:** Sylvia S. W. Tsui, Vincent W. C. Wu, Jerry S. C. Cheung

**Affiliations:** ^1^ Department of Clinical Oncology Prince of Wales Hospital Shatin Hong Kong; ^2^ Department of Health Technology & Informatics The Hong Kong Polytechnic University Hung Hom Hong Kong; ^3^ Department of Clinical Oncology Queen Mary Hospital Pok Fu Lam Hong Kong

**Keywords:** intra‐fractional error, isocenter shift, multiple brain metastases, multiple isocenters, single isocenter, SRS, SRT, stereotactic radiation therapy

## Abstract

**Introduction:**

Treatment of multiple brain metastases by linac‐based stereotactic radiotherapy (SRT) can employ either a multiple‐isocenter (MI) or single‐isocenter (SI) approach. The purposes of this study were to evaluate the dosimetric results of MI and SI approaches and compare the impacts of intra‐fractional setup discrepancies on the robustness of respective approaches using isocenter shifts, whether the same magnitude of translational and rotational effects could lead to a significant difference between the two approaches.

**Methods:**

Twenty‐two patients with multiple brain metastases treated by linac‐based SRT were recruited. Treatment plans were computed with both the MI and SI approaches. For the MI approach, the isocenter was located at the geometric center of each planning target volumes (PTVs), whereas the isocenter of the SI approach was located midway between the PTV centroids. To simulate the intra‐fractional errors, isocenter displacements including translational and rotational shifts were hypothetically applied. Apart from the dosimetric outcomes of the two approaches, the impact of the isocenter shifts on PTVs and organs at risk (OARs) were recorded in terms of the differences (*δ*) in dose parameters relative to the reference plan and was then compared between the MI and SI approaches.

**Results:**

Both MI and SI plans met the plan acceptance criteria. The mean *Paddick conformity index* (*Paddick CI*) and *D_max_* of most OARs between MI and SI plans did not show a significant difference, except that higher doses to the left optic nerve and optic chiasm were found in SI plans (*p* = 0.03). After the application of the isocenter shifts, *δCI* increased with an increase in the magnitude of the isocenter shift. When comparing between MI and SI plans, the *δCIs* were similar (*p* > 0.05) for all extents of translational shifts, but *δCIs* were significantly higher in SI plans after application of all rotations particularly ±1.5° and ±2.0° shifts. Despite the result that the majority of *δD_Max_* of OARs were higher in the SI plans, only the differences in the left optic nerve and chiasm showed generally consistent significance after both translational ≥±1 mm and rotational shifts of ≥±1${}^{\circ }$.

**Conclusion:**

Both MI and SI approaches could produce clinically acceptable plans. However, isocenter shifts brought dosimetric impacts to both MI and SI approaches and the effects increased with the increase of the shift magnitude. Although similar impacts were shown in plans of both approaches after translational isocenter shift, SI plans were relatively more vulnerable than MI plans to rotational shifts.

## 1 INTRODUCTION

Over two‐thirds of cerebral metastases were presented with multiple lesions,^1^ and these lesions have been conventionally treated by whole‐brain radiotherapy (WBRT).^2^ Currently, stereotactic radiation therapy (SRT), which utilizes hypofractionated large dose per fraction and provides highly conformal dose distribution with rapid dose fall‐off at target‐normal tissue interface, has been introduced for the treatment of brain malignancies.^1^, ^2^, ^3^ Its advantages over WBRT including improved local control have been reported in several retrospective studies.^4^, ^5^, ^6^, ^7^, ^8^ Traditionally, multiple brain lesions are treated with multiple isocenters (MIs) approach in which one isocenter is assigned for each lesion in the treatment plan. Patient repositioning and imaging sessions are required before irradiation using each isocenter, and therefore extended time is spent on setting up the treatment.^2^ The prolonged treatment time increases patient discomfort^9^ and carries the risk of patient movement and subsequently the intra‐fractional error.^10^

Alternatively, linac‐based SRT allows treatment using a single isocenter (SI) for multiple brain lesions. It provides simultaneous treatment for multiple lesions using the same setup and is able to shorten the overall treatment duration. Previous studies suggested that the SI approach could reduce the treatment time per fraction by about half^11^ in linac‐based SRT ranging from 15 to 40 min for patients having multiple brain metastases with two to ten lesions (mean = 5),^2^, ^12^ compared with the MI approach. Besides, the SI approach has been reported of being capable of achieving similar target coverage and conformity to MI plans.^11^, ^13^, ^14^, ^15^

Notably, a slightly increased dose to normal brain tissue was also associated with the SI approach when compared with that using the MI approach.^13^, ^14^, ^15^ A study by Hardcastle et al. showed that V12 Gy, which is an indicator of radiation brain necrosis, was found higher in the SI approach for multiple brain lesions.^16^ The reason may be attributed to the wider spread of low dose spillage.^3^, ^14^ Furthermore, controversies also exist as it is not certain whether the SI approach is more vulnerable to intra‐fractional setup errors. The intra‐fractional setup discrepancy is defined as the maximum difference of patient positioning between the start and the end of each fraction of treatment. Examples of intra‐fractional errors include patient motion when the stereotactic face mask does not completely immobilize the head^17^, ^18^, ^19^, ^20^; residual errors and uncertainties in robotic couch correction^21^, ^22^, ^23^, ^24^; detection errors to brain lesions based on bony anatomy^23^, ^25^; X‐ray image registration and sharp edge errors due to computed tomography (CT) slice thickness^10^, ^18^, ^19^, ^24^; and possible “counteraction” of patients after couch correction is performed.^26^ It has been reviewed that in SRT, small positional discrepancies might change and shift the overall dose distribution, substantially reducing the dose conformity of the targets as a result of the steep dose gradient.^10^, ^27^, ^28^ The difference in the extent of dosimetric impact brought by intra‐fractional shifts to MI and SI approaches has not been fully evaluated.

Therefore, the purposes of this study were to evaluate the dosimetric results of SI and MI approaches in the treatment of multiple brain metastases in linac‐based SRT, and compare the impacts of intra‐fractional setup discrepancies on the robustness of the respective approach, whether the same magnitude of translational and rotational effects could lead to a significant difference between the two approaches.

## 2 METHODS AND MATERIALS

### 2.1 Study design

Twenty‐two patients with multiple brain metastases (two to three lesions per patient) treated between 2011 and 2018 by SRT were retrospectively recruited. Patient and tumor characteristics are summarized in Table 1. Ethics approval was obtained from the hospital concerned, and all the patient data were pseudonymized. These patients were scanned with a CT simulator in a treatment position with a non‐invasive frameless‐based stereotactic system thermoplastic mask (BrainLAB Frameless Radiosurgery Mask) for immobilization. Both non‐contrast and contrast CT images with 1–1.5‐mm slice thickness were acquired for treatment planning. Magnetic resonance imaging with 1.5‐mm thickness was also taken for delineation of target volumes after registration with the CT images. The planning target volume (PTV) was generated by adding a 1–2‐mm margin to the gross tumor volume by the responsible oncologist. Multiple brain metastatic lesions were prescribed individually at around 80% according to their size. The prescriptions were hypofractionated schedules either 7–8 Gy per fraction for three fractions or 6–7 Gy per fraction for four to five fractions.^29^, ^30^, ^31^

**TABLE 1:** Patient (*n* = 22) and tumor lesions (*n* = 46) characteristics

**Parameters**		***n* (%)**	**Mean**	**Range**
Gender:				
Male		14 (63.6%)		
Female		8 (36.4%)		
Age (year):			61	37–79
Number of lesions per patient:				
Two‐site		20 (90.9%)		
Three‐site		2 (9.1%)		
Location of metastases:				
Right (*n* = 25)	Frontal	8 (17.4%)		
	Parietal	9 (19.6%)		
	Temporal	1 (2.2%)		
	Occipital	4 (8.7%)		
	Cerebellum	3 (6.5%)		
Left (*n* = 20)	Frontal	3 (6.5%)		
	Parietal	5 (10.9%)		
	Temporal	3 (6.5%)		
	Occipital	3 (6.5%)		
	Cerebellum	5 (10.9%)		
	Cerebellopontine angle	1 (2.2%)		
Central (*n* = 1)	Cerebellar vermis	1 (2.2%)		
Planning target volume (PTV; $cm^{3}$):				
Per lesion			7.7	0.4–71.0
Per patient (i.e., $\mathrm{PT}V_{\mathrm{total}}$)			16.1	2.2–74.3

### 2.2 Treatment planning

Treatment planning was performed using the Eclipse system (Varian Medical Systems, Version 13.6). In case there were multiple targets with different prescriptions in the same plan, the dose prescriptions of individual PTVs would be amended to their relative dose at 100% isodose level for plan computation. Dose distributions were then generated for every target accordingly. All the plans were calculated using the anisotropic analytical algorithm (AAA) in Eclipse. The grid resolution of 2 mm was applied normally. For very small targets with a diameter less than 1 cm, it might be reduced to 1 mm.

Regarding the MI approach, the number of isocenter per patient is equivalent to the number of brain metastases. The isocenter was located at the geometric center of the respective PTV.^14^, ^16^ Different isocenters were allocated to distinct PTVs in the same plan. Multiple coplanar and non‐coplanar static intensity‐modulated radiation therapy (IMRT) beams with 6‐MV photons from linear accelerator equipped with Brainlab M3 micro‐multileaf collimators (micro‐MLCs) were used. The total number of beams used for each patient case ranged from 12 to 21 (average 13.5 beams) and the average number of beams per target was 7.0. The setting of the dose constraints for organs at risk (OARs), which included the brainstem, optic nerves, optic chiasm, and eyes, was in accordance with the American Association of Physicists in Medicine Task Group 101 (AAPM TG 101)^32^ and the United Kingdom the Royal College of Radiologists (RCR).^33^ The acceptance criteria for targets were that at least 98% of PTV was covered by the prescribed dose with maximized *Paddick CI* achieved. By definition, the *CI* ranges from 0 to 1.0 based on the guidelines of the International Commission on Radiation Units and Measurements (ICRU) for stereotactic radiosurgery (SRS)^34^, ^35^ (ideal *CI* is 1.0; plan quality decreases with decreasing index).

While for the SI approach, an isocenter was located roughly midway between the centroids of all the PTVs with each target weighted equally. For each patient, the SI plan was generated using six to nine non‐coplanar static IMRT beams (most commonly seven beams) with the same radiation energy, target volumes, OARs, and their corresponding dose constraint requirements as of the MI plan. The gantry and couch angles were chosen depending on the locations of OARs and PTVs so that the beams could avoid or minimize direct irradiation to OARs and reduce the radiation path from the skin surface to PTVs. The collimator angles were adjusted based on the shape of the targets so that the optimum MLC pattern could be utilized.

A summary of both MI and SI plans indicating all the PTV volumes, number of beams used per plan, and the respective *CI*s obtained is attached in the Appendix.

### 2.3 Simulation of intra‐fractional errors using isocenter shifts

To simulate the intra‐fractional errors, isocenter displacements were hypothetically applied to plans of both approaches. The displacements included three translational directions as antero‐posterior (AP), left‐right (LR), and supero‐inferior (SI) directions and three rotational dimensions including roll, yaw, and pitch. Both original plans of MI and SI approaches were replicated to new plans using the same treatment parameters but with different shifted‐isocenter directions or CT volume rotations. New doses for analysis were then obtained after recalculation. A total of 16 types of new plans were generated for each original plan, including translational shifts of +0.5, –0.5, +1, –1, +1.5, –1.5, +2, –2 mm (every 0.5 mm increment from –2 to +2 mm) in each of the LR, AP, and SI directions and rotation shifts of +0.5${}^{\circ }$, –0.5${}^{\circ }$, +1${}^{\circ }$, –1${}^{\circ }$, +1.5${}^{\circ }$, –1.5${}^{\circ }$, +2${}^{\circ }$, –2${}^{\circ }$ (every 0.5${}^{\circ }$ increment from –2${}^{\circ }$ to +2${}^{\circ }$) for each of the roll, yaw, and pitch directions (+ and – sign represented opposite directions). The reason to set the maximum shifts to 2 mm and 2${}^{\circ }$ was that most setup deviations in SRS/SRT fall within these ranges. Mean intra‐fractional errors were usually reported to be within 1 mm for translation and 1${}^{\circ }$ for rotation by several studies.^10^, ^17^, ^18^, ^36^, ^37^ Just a small number of patients experienced > 2‐mm fluctuations,^17^ and significant dose effects were observed in PTV coverage only when a 2.0${}^{\circ }$ rotational error was simulated using the SI technique.^28^ The extent of shifts employed in this study could also be referenced from a study by Prentou et al.^38^

As to study the maximal effect of translational or rotational errors, the shifts of the same magnitude in three directions (translation or rotation) were applied simultaneously for every isocenter in each new plan. For better illustration, a summary of all the shift combinations is given in Table 2.

**TABLE 2:** Summary of all the hypothetical isocenter shift combinations for multiple isocenter (MI) and single isocenter (SI) approaches

	**Translational shift (mm)**			**Rotational shift (** ${}^{\circ }$)		
**Types of plans**	**LR**	**SI**	**AP**	**Yaw**	**Roll**	**Pitch**
1	+0.5	+0.5	+0.5			
2	+1.0	+1.0	+1.0			
3	+1.5	+1.5	+1.5			
4	+2.0	+2.0	+2.0			
5	–0.5	–0.5	–0.5			
6	–1.0	–1.0	–1.0			
7	–1.5	–1.5	–1.5			
8	–2.0	–2.0	–2.0			
9				+0.5${}^{\circ }$	+0.5${}^{\circ }$	+0.5${}^{\circ }$
10				+1.0${}^{\circ }$	+1.0${}^{\circ }$	+1.0${}^{\circ }$
11				+1.5${}^{\circ }$	+1.5${}^{\circ }$	+1.5${}^{\circ }$
12				+2.0${}^{\circ }$	+2.0${}^{\circ }$	+2.0${}^{\circ }$
13				–0.5${}^{\circ }$	–0.5${}^{\circ }$	–0.5${}^{\circ }$
14				–1.0${}^{\circ }$	–1.0${}^{\circ }$	–1.0${}^{\circ }$
15				–1.5${}^{\circ }$	–1.5${}^{\circ }$	–1.5${}^{\circ }$
16				–2.0${}^{\circ }$	–2.0${}^{\circ }$	–2.0${}^{\circ }$

Abbreviations: AP, antero‐posterior; LR, left‐right; SI, supero‐inferior.

### 2.4 Analysis of treatment plans

Each treatment plan was evaluated by collecting the dose information of all the target volumes and OARs. The dose coverage of PTV was evaluated by *CI* and “*volume of regret*” (*VoR*).

The *CI* was calculated using the formula advocated by Paddick^39^ and is defined as

(1) $$CI_{Paddick}\;=\;\frac{T{V_{PIV}}^{2}}{TV\;\times PIV}$$

where *TV* is the target volume, *TV_PIV_* is the volume of *PTV* covered by the prescribed dose and *PIV* is the total volume covered by the prescribed dose. Since multiple targets were presented within one single plan for both MI and SI techniques, attention was put to obtain the value of *PIV*, in which a *PIV* was obtained for each target volume rather than a *PIV* for the whole plan. A unique *CI* value that was unaffected by the dose impact of other PTVs could thus be computed for each target. The perfect conformity is represented as *CI* value of 1.0.

While for the *VoR*, it was calculated as the percentage of PTV volume not covered by the prescribed dose. Four levels of *VoR* were identified which included ≤2%, 2%–5%, 5%–10%, and ≥10%. The incidence (% of plans) of each level of *VoR* under different magnitudes of isocenter shifts was hence compared between MI and SI approaches.

In addition, the differences of dose parameter (Change of *CI* (*δCI*) for PTVs and Change of Dose Maximum (*δD_max_*) for OARs doses) due to isocenter shift were calculated as the absolute value of the difference between the *CI* of the individual PTV/*D_max_* of individual OAR in the new plan (P_NP_) and that in the reference plan (P_RP_, which is the original plan with no isocenter shift). The *δCI* and *δD_max_* for each extent of the shift were examined between the two approaches as well.

All data were analyzed using SPSS Statistics Version 22 software (IBM Corp). Paired *t‐*test was employed to evaluate the differences

## 3 RESULTS

### 3.1 Plan comparison between MI and SI approaches

**FIGURE 1:**
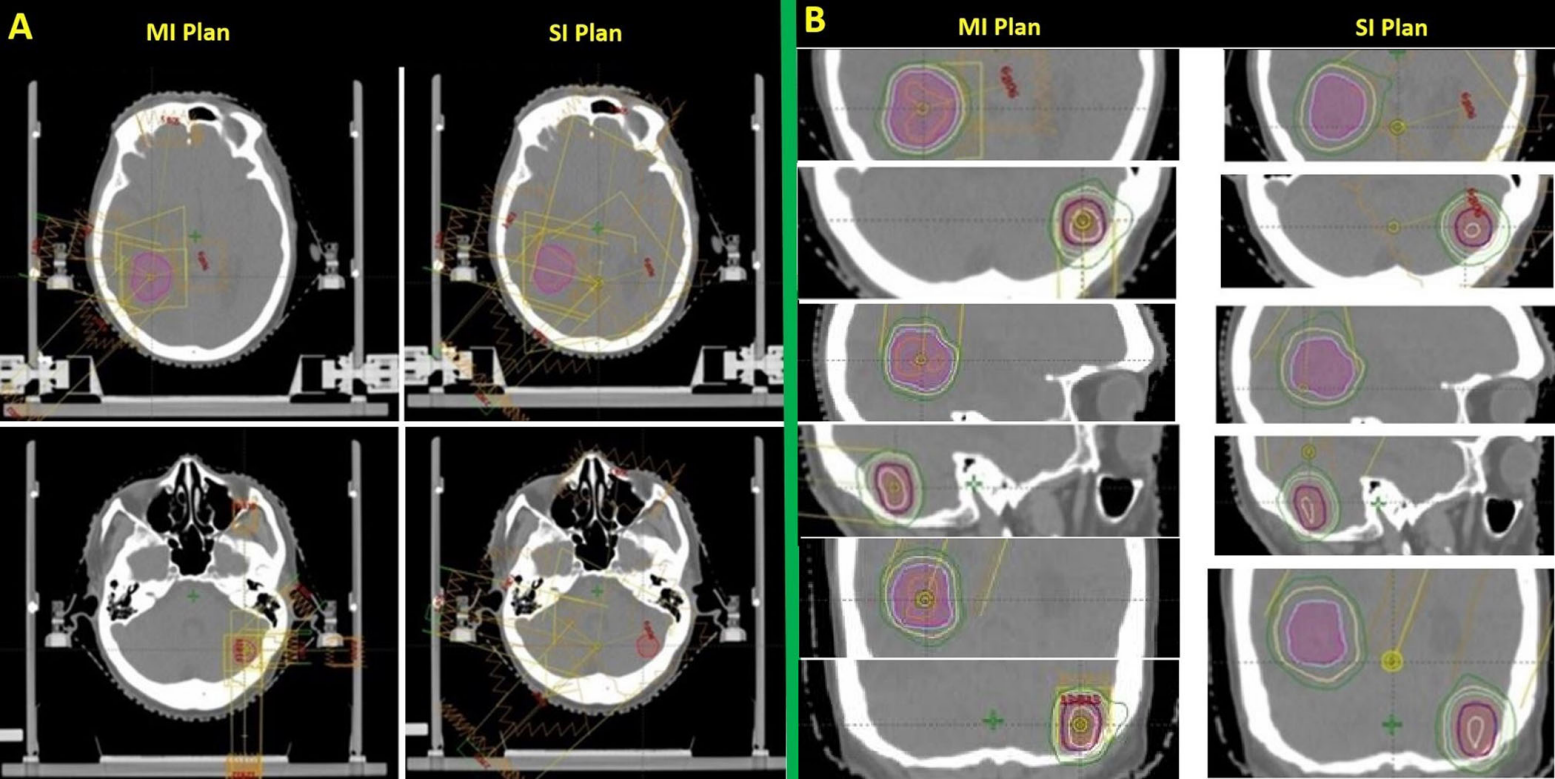
Illustration of multiple isocenter (MI) and single isocenter (SI) treatment plans on a patient with two brain lesions treated by stereotactic radiosurgery radiotherapy. (a) The beam arrangements, (b) the dose distribution of individual targets in axial, coronal, and sagittal planes of the respective approaches

A total of 46 lesions from 22 subjects were evaluated. Two patients were treated with three brain lesions, and the rest were treated with two lesions. An example of the treatment plan demonstrating the beam arrangements and dose distribution by MI and SI approaches is shown in Figure 1a,b, and a summary of dose parameters of PTVs and OARs for all patients is shown in Table 3. The mean *CI* of SI plans (0.83) was comparable to that of the MI plans (0.84; *p* = 0.261). For the doses to OARs, the majority of them showed higher mean maximum doses (*D_max_*) in the SI plans relative to that in MI plans except for the brainstem. Among them, the differences in doses of the left optic nerve and optic chiasm reached statistical significance (*p* = 0.03).

**TABLE 3:** Summary of dose parameters of PTV and organs at risk (OARs) in plans using MI and SI approaches

		**MI plan**	**SI plan**		**Dose tolerances** **^#^**	
				
**Structure**	**Dose parameter**	**Mean ± SD**		***p*‐value**	**Threshold dose (Gy)**	
PTV						
	Conformity index	0.84 ± 0.06	0.83 ± 0.06	0.261		
OARs					Three fractions	Five fractions
Brainstem	D_max_ (Gy)	7.98 ± 9.94	6.69 ± 7.32	0.25	18	23
Optic nerve (R)	D_max_ (Gy)	1.23 ± 3.68	1.63 ± 3.01	0.07	15	22.5
Optic nerve (L)	D_max_ (Gy)	1.40 ± 2.39	2.29 ± 3.03	**0.03**	(For the total optic pathway)	
Chiasm	D_max_ (Gy)	2.43 ± 3.80	3.51 ± 4.05	**0.03**		
Eye (R)	D_max_ (Gy)	0.61 ± 1.10	0.66 ± 0.76	0.76	8*	8*
Eye (L)	D_max_ (Gy)	0.93 ± 1.70	1.09 ± 1.70	0.43	8*	8*

Abbreviations: D_max_, maximum dose; Gy, Gray; L, left; MI, multiple isocenter; R,right; SI, single isocenter.

^#^ Dose tolerances of different normal tissues for stereotactic body radiation therapy according to AAPM TG 101 by Benedict et al.^32^ and the UK RCR consensus by Hanna et al.^33^ ^.^

* Orbit was selected as a surrogate for retina. Its constraint is not specifically designed for three/five fractions of treatment. Instead, 8 Gy, where it is the optimal threshold dose for a single fraction, is shown here.

### 3.2 Comparison on the impact of isocenter shift between MI and SI approaches

In this part, the original plans with no isocenter shift were regarded as reference plans that met the target and OAR dose acceptance criteria.

**FIGURE 2:**
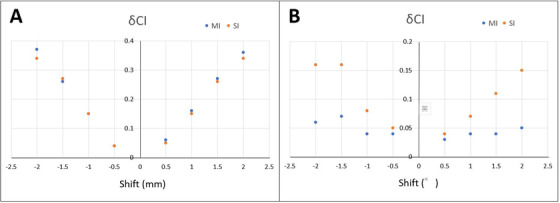
Comparison of *δCI* with respect to the impacts of different extents of (a) translational isocenter shifts and (b) rotational isocenter shifts to planning target volumes (PTVs) between MI and SI plans

**FIGURE 3:**
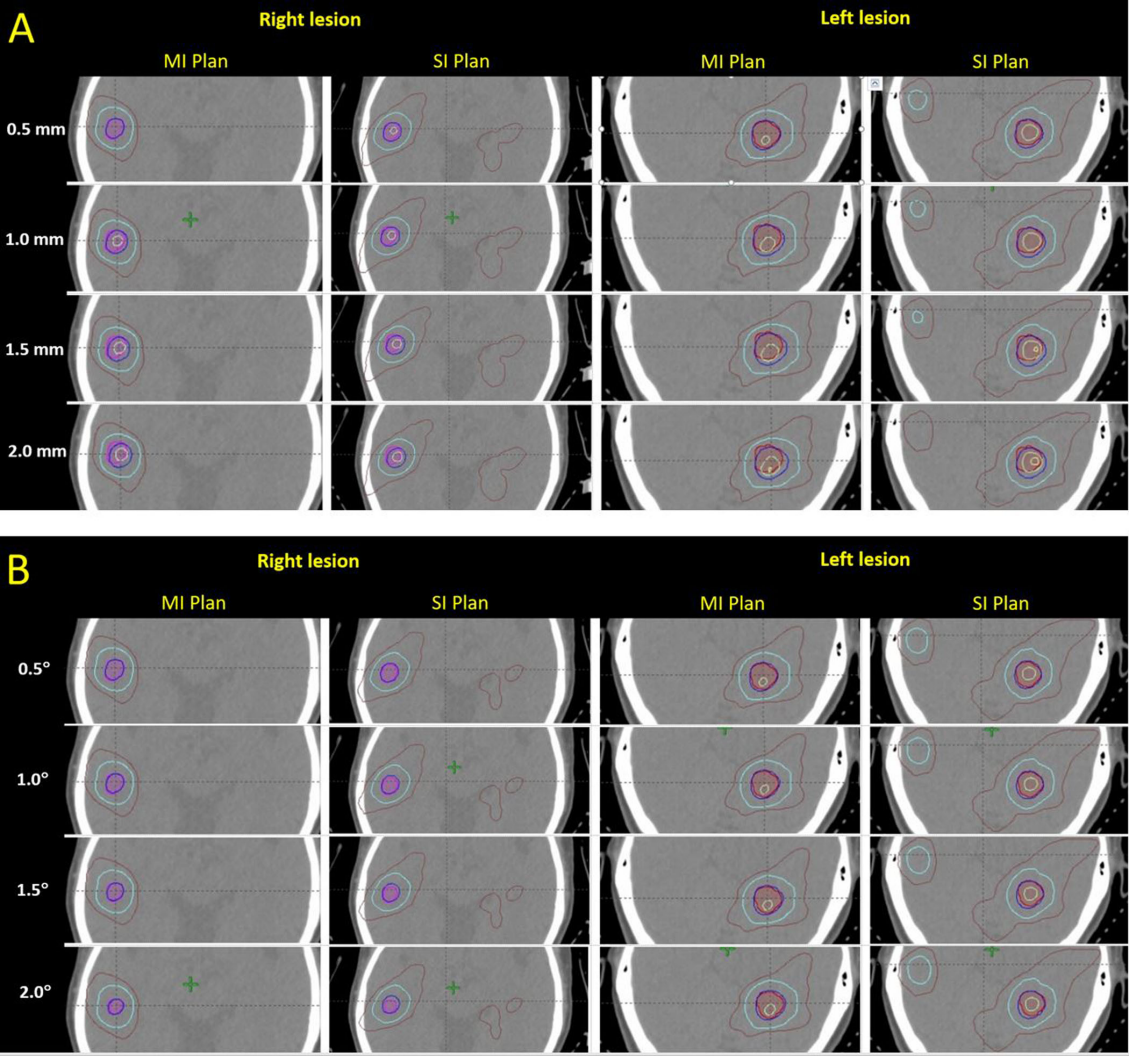
Comparison of the target (right and left brain lesions) dose distributions in axial computed tomography planes under different magnitudes of (a) translational isocenter shifts and (b) rotational isocenter shifts between MI and SI plans. Red line/pink line = PTV, blue line = prescribed isodose level (80%)

With regard to the PTV, *δCI* for all magnitudes of translational shifts were similar between the MI and SI plans (*p *> 0.05; Table 4) (Figure 2a). Instead, for the rotational shifts, the SI plans showed significantly greater *δCI* than that of MI plans after all extents of shifts (*p* ≤ 0.05; Table 5; Figure 2b), with the differences being exaggerated particularly after ±1.5° and ±2.0° shifts. Illustrations of the effect of translational and rotational isocenter shifts on target dose distributions are shown in Figure 3a,b. The figure displays that the deviations between the prescribed dose line (blue) and the PTV target (red/pink) were further magnified after 1.5${}^{\circ }$ and 2${}^{\circ }$ shifts when compared between the two approaches. In general, *δCI* of SI plans increased when the magnitude of rotational shift increased, whereas that of MI plans could be kept more stable for all magnitudes of rotational shifts. Another noteworthy point is that translational isocenter shifts brought greater influence than rotational shifts in both MI and SI approaches; and this was reflected by the larger magnitude of *δCI*s in translational shifts (Tables 4 and 5).

**TABLE 4:** Comparison of the effects of translational isocenter shifts (+0.5, ‐0.5, +1, –1, +1.5, –1.5, +2, –2 mm) on MI and SI plans

	**Change of CI (δCI)**			**Change of D_max_ (δD_max_) (Gy)**																		
	**PTV**			**Brainstem**			**R‐optic nerve**			**L‐optic nerve**			**Chiasm**			R‐eye			L‐eye		
**Shift (mm)**	**MI**	**SI**	***p***	**MI**	**SI**	***p***	**MI**	**SI**	***p***	**MI**	**SI**	***p***	**MI**	**SI**	***p***	**MI**	**SI**	***p***	**MI**	**SI**	***p***
+0.5	0.06	0.05	*0.579*	0.21	0.21	0.970	0.03	0.12	0.153	0.06	0.15	0.230	0.07	0.10	0.397	0.02	0.02	0.911	0.04	0.07	0.400
−0.5	0.04	0.04	*0.598*	0.23	0.25	0.507	0.03	0.11	0.166	0.06	0.14	0.254	0.07	0.10	0.750	0.02	0.02	0.862	0.03	0.08	0.218
+1.0	0.16	0.15	*0.340*	0.17	0.42	0.185	0.03	0.23	0.103	0.05	0.31	**0.004**	0.04	0.24	**0.002**	0.01	0.03	0.157	0.04	0.13	0.086
–1.0	0.15	0.15	*0.977*	0.21	0.48	0.237	0.04	0.27	0.093	0.06	0.24	**0.014**	0.04	0.21	**0.011**	0.01	0.03	0.328	0.04	0.19	**0.024**
+1.5	0.27	0.26	*0.337*	0.22	0.61	0.135	0.05	0.34	0.109	0.07	0.46	**0.004**	0.06	0.33	**0.002**	0.02	0.05	0.112	0.05	0.19	0.052
–1.5	0.26	0.27	*0.869*	0.31	0.67	0.252	0.05	0.20	0.297	0.07	0.37	**0.012**	0.07	0.37	**0.011**	0.02	0.04	0.421	0.06	0.33	**0.014**
+2.0	0.36	0.34	*0.075*	0.29	0.73	0.171	0.06	0.44	0.130	0.09	0.67	**0.005**	0.08	0.44	**0.004**	0.02	0.06	0.116	0.07	0.23	0.078
–2.0	0.37	0.34	*0.066*	0.39	0.88	0.227	0.07	0.38	0.070	0.10	0.50	**0.004**	0.09	0.53	**0.009**	0.03	0.05	0.460	0.08	0.49	**0.017**

δCI = $|\mathrm{Paddick}\;\mathrm{CI}\;\mathrm{of}\;\mathrm{PTV}\;\mathrm{in}\;\mathrm{new}\;\mathrm{plan}\;\mathrm{after}\;\mathrm{isocenter}\;\mathrm{shift}\;\left(P_{\mathrm{NP}}\right)\;-\;\mathrm{CI}\;\mathrm{of}\;\mathrm{that}\;\mathrm{PTV}\;\mathrm{in}\;\mathrm{reference}\;\mathrm{plan}\;\left(P_{\mathrm{RP}},\;\mathrm{which}\;\mathrm{is}\;\mathrm{the}\;\mathrm{original}\;\mathrm{plan}\;\mathrm{with}\;\mathrm{no}\;\mathrm{shift}\right)|.$

δD_max _= $|\mathrm{Maximum}\;\mathrm{dose}\;\left(\mathrm{Dmax}\right)\mathrm{of}\;\mathrm{OAR}\;\mathrm{in}\;\mathrm{new}\;\mathrm{plan}\;\mathrm{after}\;\mathrm{isocenter}\;\mathrm{shift}\;\left(P_{\mathrm{NP}}\right)\;-\;\mathrm{Dmax}\;\mathrm{of}\;\mathrm{that}\;\mathrm{OAR}\;\mathrm{in}\;\mathrm{reference}\;\mathrm{plan}\;\left(P_{\mathrm{RP}},\;\mathrm{which}\;\mathrm{is}\;\mathrm{the}\;\mathrm{original}\;\mathrm{plan}\;\mathrm{with}\;\mathrm{no}\;\mathrm{shift}\right)|.$

Abbreviations: MI, multiple isocenter; SI, single isocenter.

**TABLE 5:** Comparison of the effects of rotational isocenter shifts (+0.5°, –0.5°, +1°, –1°, +1.5°, –1.5°, +2°, –2°) on MI and SI plans

	**Change of CI (δCI)**			**Change of D_max_ (δD_max_) (Gy)**																	
	**PTV**			**Brainstem**			**R‐optic nerve**			**L‐optic nerve**			**Chiasm**			**R‐eye**			**L‐eye**		
**Shift (degree)**	MI	**SI**	***p***	**MI**	**SI**	***p***	**MI**	**SI**	***p***	**MI**	**SI**	***p***	**MI**	**SI**	***p***	**MI**	**SI**	***p***	**MI**	**SI**	***p***
+0.5°	0.03	0.04	***0.030***	0.04	0.12	0.629	0.04	0.07	0.498	0.21	0.11	0.813	0.13	0.09	0.051	0.04	0.02	0.884	0.17	0.12	0.366
–0.5°	0.04	0.05	***0.017***	0.05	0.07	0.710	0.08	0.04	**0.010**	0.15	0.14	0.988	0.16	0.09	0.134	0.04	0.01	0.887	0.11	0.12	0.929
+1.0°	0.04	0.07	***0.001***	0.10	0.17	0.388	0.02	0.16	0.063	0.04	0.28	**0.006**	0.02	0.16	**0.005**	0.02	0.03	0.529	0.06	0.22	0.152
–1.0°	0.04	0.08	***0.001***	0.21	0.16	0.706	0.04	0.07	0.576	0.06	0.26	**0.025**	0.05	0.16	0.061	0.02	0.03	0.461	0.04	0.22	**0.024**
+1.5°	0.04	0.11	***0.001***	0.11	0.31	0.080	0.03	0.20	0.057	0.06	0.38	**0.018**	0.04	0.21	**0.009**	0.03	0.04	0.555	0.09	0.32	0.177
–1.5°	0.07	0.16	***0.050***	0.11	0.27	0.182	0.03	0.13	0.223	0.06	0.32	**0.013**	0.05	0.25	**0.021**	0.02	0.04	0.431	0.07	0.40	**0.016**
+2.0°	0.05	0.15	***0.001***	0.11	0.40	**0.044**	0.04	0.30	**0.044**	0.07	0.46	**0.022**	0.05	0.31	0.146	0.04	0.05	0.561	0.12	0.37	0.231
–2.0°	0.06	0.16	***0.001***	0.14	0.40	0.088	0.04	0.25	0.085	0.06	0.45	**0.015**	0.06	0.35	**0.023**	0.03	0.05	0.425	0.08	0.60	**0.011**

δCI = $|\mathrm{Paddick}\;\mathrm{CI}\;\mathrm{of}\;\mathrm{PTV}\;\mathrm{in}\;\mathrm{new}\;\mathrm{plan}\;\mathrm{after}\;\mathrm{isocenter}\;\mathrm{shift}\;\left(P_{\mathrm{NP}}\right)\;-\;\mathrm{CI}\;\mathrm{of}\;\mathrm{that}\;\mathrm{PTV}\;\mathrm{in}\;\mathrm{reference}\;\mathrm{plan}\;\left(P_{\mathrm{RP}},\;\mathrm{which}\;\mathrm{is}\;\mathrm{the}\;\mathrm{original}\;\mathrm{plan}\;\mathrm{with}\;\mathrm{no}\;\mathrm{shift}\right)|$.

δD_max _= $|\mathrm{Maximum}\;\mathrm{dose}\;\left(\mathrm{Dmax}\right)\mathrm{of}\;\mathrm{OAR}\;\mathrm{in}\;\mathrm{new}\;\mathrm{plan}\;\mathrm{after}\;\mathrm{isocenter}\;\mathrm{shift}\;\left(P_{\mathrm{NP}}\right)\;-\;\mathrm{Dmax}\;\mathrm{of}\;\mathrm{that}\;\mathrm{OAR}\;\mathrm{in}\;\mathrm{reference}\;\mathrm{plan}\;\left(P_{\mathrm{RP}},\;\mathrm{which}\;\mathrm{is}\;\mathrm{the}\;\mathrm{original}\;\mathrm{plan}\;\mathrm{with}\;\mathrm{no}\;\mathrm{shift}\right)|$.

Abbreviations: MI, multiple isocenter; SI, single isocenter.

Similar results were observed in the *VoR* in which there were increases in the incidence of *VoR* with the increase in magnitude after any types of isocenter shifts, and the percentage of plans resulted in higher *VoR* levels (5%–10% and ≥10%) in the translational shifts and were more than those of the rotational shifts for both MI and SI approaches (Table 6). Comparable results among MI and SI plans were found regarding the respective magnitude of translational isocenter shift as similar percentage of plans were observed in each *VoR* level. In the case of rotational shifts, the majority of plans using the MI approach could result in *VoR* ≤2% for all extents of shifts. Yet plans using the SI approach appeared more in *VoR* of 5%–10% and ≥10% along with increased magnitude of shifts. More obvious differences between MI and SI approaches were identified in the rotational shifts. These results were consistent with the results from the above part using *δCI* for analysis.

**TABLE 6:** Comparison of the incidence (in %) with different levels of “*volume of regret*” (*VoR*) between MI and SI plans

		**VoR**							
		**≤ 2%**		**2%–5%**		**5%–10%**		**≥10%**	
		**MI plan**	**SI plan**	**MI plan**	**SI plan**	**MI plan**	**SI plan**	**MI plan**	**SI plan**
**Shift**	**Magnitude**	**(%Plan)**	**(%Plan)**	**(%Plan)**	**(%Plan)**	**(%Plan)**	**(%Plan)**	**(%Plan)**	**(%Plan)**
Translational	+0.5 mm	23.9	34.8	45.7	37.0	28.3	26.1	2.2	2.2
	–0.5 mm	21.7	19.6	41.3	34.8	32.6	39.1	4.3	6.5
	+1 mm	0	0	17.4	19.6	28.3	30.4	54.3	50.0
	–1 mm	0	0	8.7	13.0	26.1	26.1	65.2	60.9
	+1.5 mm	0	0	6.5	4.3	17.4	21.7	76.1	73.9
	–1.5 mm	0	0	0	0	13.0	15.2	87.0	84.8
	+2 mm	0	0	0	0	8.7	8.7	91.3	91.3
	–2 mm	0	0	0	0	2.2	0	97.8	100.0
									
Rotational	+0.5${}^{\circ }$	93.5	69.6	4.3	28.3	2.2	2.2	0	0
	–0.5${}^{\circ }$	97.8	82.6	2.2	15.2	0	2.2	0	0
	+1.0${}^{\circ }$	89.1	45.7	10.9	30.4	0	17.4	0	6.5
	–1.0${}^{\circ }$	100.0	52.2	0	26.1	0	15.2	0	6.5
	+1.5${}^{\circ }$	95.7	32.6	4.3	23.9	0	21.7	0	21.7
	–1.5${}^{\circ }$	100.0	39.1	0	28.3	0	15.2	0	17.4
	+2.0${}^{\circ }$	87.0	19.6	13.0	30.4	0	13.0	0	37.0
	–2.0${}^{\circ }$	91.3	23.9	8.7	32.6	0	10.9	0	32.6

VoR = % volume of PTV not covered by prescribed isodose.

%Plan = number of plans/total number of plans x 100%.

**FIGURE 4:**
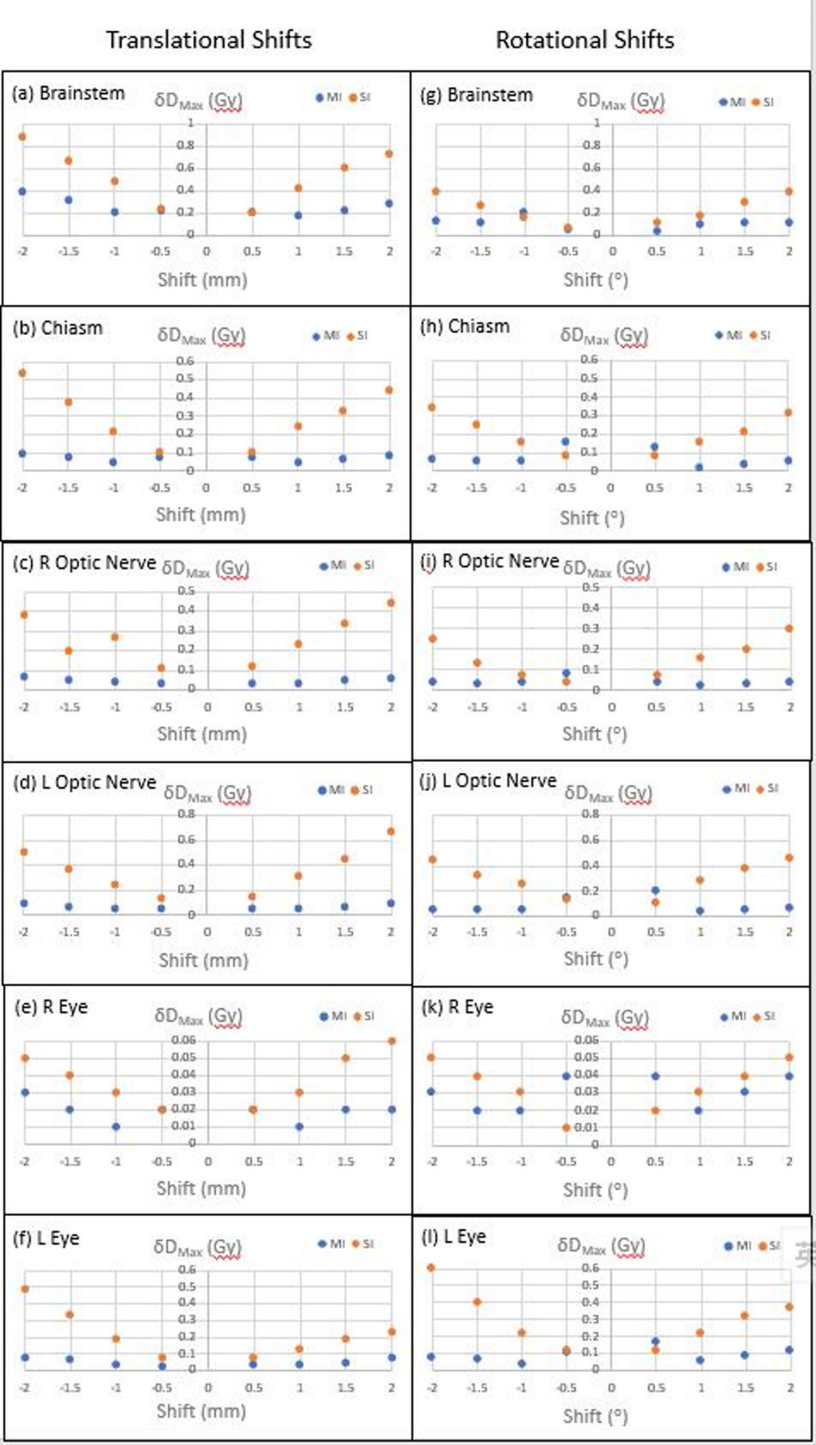
Comparison of *δD_Max_* with respect to the impacts of different extents of (a‐f) translational isocenter shifts and (g‐l) rotational shifts to respective organs at risk between MI and SI plans. L = left, R = right

For the OARs, concerning the translational shifts, most *δD_Max_* of doses of OARs in SI plans were greater than those of the MI plans. This difference between SI and MI plans grew with increasing magnitude of shift (Figures 4a‐f); however, these differences were relatively small and did not reach statistical significance for the brainstem, right optic nerve, and both sides of eyes (Table 4). The differences became significant for the left optic nerve and chiasm after shifts ≥±1.0 mm (*p* ≤ 0.02). For both approaches, generally, the magnitude of respective *δD_Max_* of different OARs doses was also shown with an upward trend when the extent of the translational shift was enlarged. Only the *δD_Max_* of brainstem doses in SI plans after ±1.5 mm and ±2 mm shift was greater than 0.6 Gy (Figure 4a) but that for the majority of other OARs doses were less than 0.4 Gy in SI plans and even less than 0.1 Gy in MI plans for all types of translational shifts.

Regarding the influence of rotational shifts, a larger portion of *δD_Max_* of OARs doses in SI plans was greater than those of the MI plans (Figure 4g–l), with only a few differences of OARs doses revealed as statistically significant (*D_max_* of brainstem after +2${}^{\circ }$ shift; right optic nerve after –0.5${}^{\circ }$ and +2${}^{\circ }$ shift; left optic nerve after ≥±1${}^{\circ }$ shift; chiasm after +1${}^{\circ }$, ±1.5${}^{\circ },$ and – 2${}^{\circ }$ shift; left eye after ≥ –1${}^{\circ }$ shift; Table 5). Similar to the impacts of translational shifts to OARs doses, extreme values of shifts (≥ ±1.5${}^{\circ }$) have resulted in wider deviations in *δD_Max_* between the two planning approaches. Additionally, in the SI plans, the trend was demonstrated as the larger the magnitude of rotational shifts, the higher the *δD_Max_* of OARs doses. All *δD_Max_* were less than 0.3 Gy and 0.6 Gy for MI and SI approach after all extents of rotational shifts, respectively.

## 4 DISCUSSION

### 4.1 Quality of plans for MI and SI approaches

In terms of target conformity and doses to OARs, both MI and SI approaches produced clinically acceptable SRT plans for patients with multiple brain lesions. These results were in line with previous studies^3^, ^15^ that reported that the SI approach produced comparable target dose coverage and normal tissue doses when compared with the MI approach in SRT of multiple brain metastases. Algan et al. also demonstrated that there was an added advantage of SI plans that was a 35% reduction in beam‐on time.^15^

Although both approaches could produce plans satisfying the plan requirements, we found that the SI plans would result in relatively lower PTV dose conformity (*CI* of 0.83 vs. 0.84 but not statistically significant) and higher maximum doses to some OARs (e.g., left optic nerve and chiasm; significantly higher doses) than the MI plans. The relatively poorer target conformity in SI plans could be explained by the fact that better target conformity could be achieved with a greater number of isocenters and radiation beams^40^ as in the case of the MI plans. While one isocenter was assigned to each target in the MI plans in which the dose distribution for each target could be adjusted independently, for the SI plans, the isocenter was placed at roughly midway between targets, and the planning was performed by considering all targets together. These inevitably limited the flexibility of manipulating the treatment parameters of individual targets and therefore would lead to a less ideal target dose distribution in SI plans. Moreover, Morrison et al. reported that target conformity and gradient indices worsen with increasing distance of the isocenter from the PTVs.^3^ The brain lesions were usually sparsely located. The location where the isocenter was placed in SI plans may be within the normal brain tissue, when compared with isocenters in MI plans which were placed inside each brain lesion, or even at the center of each brain mass. Planning with a SI for multiple brain lesions usually encounters disadvantages brought by a larger distance of isocenter with PTVs.

A possible explanation for relatively higher doses received by a few OARs in SI plans than in MI plans is that larger collimator size and the use of wider MLCs were usually required in the SI plans to cover all targets in linac‐based SRT. This would reduce the ability to shape the dose around the target and at the same time avoid the dose to different OARs. Besides, it would also lead to more leakage dose between MLCs and greater scattered radiation,^41^ hence subsequently increased the doses to OARs. An island blocking problem would occur, when multiple targets (≥2) share the same pair of MLC, causing an area of non‐target tissue that is not covered by the MLCs.^14^

Nevertheless, since the overall dosimetric differences between MI and SI approaches were relatively subtle, most researchers advocated that the effect was clinically insignificant.^3^, ^40^ This has been supported by several clinical studies in which the local control and toxicities were comparable between these two approaches.^1^, ^12^, ^42^ Lau et al. only deduced that minor improvements in plan quality can be attained by MI.^1^ In addition to the advantage of the resulting shorter treatment time, the SI approach in the treatment of multiple brain lesions was generally appreciated by oncology departments.

### 4.2 Effects of isocenter shifts on treatment plans

After the introduction of isocenter shifts that aimed to simulate the intra‐fractional setup discrepancies in the daily clinical situation, the PTV doses were all affected in both MI and SI plans. The result in this study illustrated larger effects with translational shifts than with rotational shifts according to the magnitude of *δCI* in both plans. This echoed the report from Wang et al. who studied the dosimetric results in spinal stereotactic body radiotherapy and addressed that a 2‐mm translational error could result in > 5% tumor coverage loss and > 25% maximal dose increase to OARs.^27^ The lower dosimetric impact in the rotational shifts could be due to the relatively small tumor volumes in SRT and the geometrical relationship between the isocenter(s) and PTVs. Extreme cases were found after ±1.5 mm translational shifts, where *δCI*s exceeded 0.25, and ±2 mm translational shifts, where *δCI*s were greater than 0.33 (Table 4), and the percentage of plans having *VoR* of ≥10% was greater than 91% in both approaches (Table 6). This revealed that large magnitude of translational shifts degraded the dose coverage and conformity to PTV and would result in non‐clinically acceptable plans. In contrast, there would be less concern in deterioration in PTV doses for rotational shifts within ±2° since all *δCI*s were ≤ 0.16 for the SI approach or even < 0.1 in MI plans (Table 5).

With respect to the comparison of the impact of the isocenter shift on PTV dose between the MI and SI approaches, no statistically significant differences in *δCI* were found for all extents of translational shifts in the study. It is suggested that the geometrical relationship of the shifted‐dose distribution and isocenter were moved in the same way for both MI and SI planning. Translational shifts do not appear to affect MI and SI plans differently. A larger extent of shifts resulted in further loss in *CI* when compared with its original plan (without shift) but affected SI and MI plans similarly.

**FIGURE 5:**
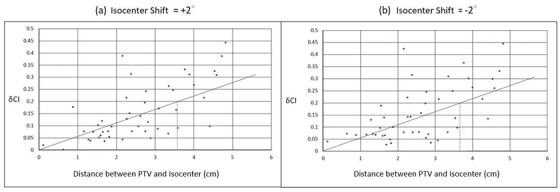
Scattered plots showing the relationship of *δCI* against PTV‐isocenter distance in SI plans after application of rotational shifts of (a) +2${}^{\circ }$ and (b) –2${}^{\circ }$

Yet regarding the rotational shifts, SI plans were in general relatively more vulnerable than the MI plans as significant differences in *δCI*s were found after all magnitudes of shifts. This was reflected in the values of *δCI*s and analysis of *VoR*s, especially for 5%–10% and ≥10% of *VoR*s when shifts ≥ ±1.5${}^{\circ }$. The main reason for this lies in the difference in the PTV‐isocenter relationship.^43^ The isocenter was the center of rotation where it was placed at the center of the PTVs in MI plans, whereas the isocenter for SI plans was distant from the PTVs in view of covering more than one target. Any shift would bring greater dose changes in PTVs than the MI plans. The effect would be magnified for PTVs situated further away from the isocenter. This observation can be further illustrated in the scattered plots (Figure 5a,b) demonstrating the relationship between the distance of isocenter from PTVs and the relative degree of change of target dose conformity (*δCI*) in SI plans when the rotational shifts were +2.0${}^{\circ }$ and –2${}^{\circ },$ respectively. PTV‐isocenter distances were calculated using the root mean square of the differences in LR (lateral; *x*), AP (vertical; *y*), and SI (longitudinal; *z*) directions between the target PTV and its respective isocenter location, specifically as

(2) $$\begin{array}{ccc} & & \mathrm{Distance}\;\mathrm{of}\;\mathrm{isocenter}\;\mathrm{from}\;\mathrm{PTV}\;\\ & & \;=\;\;\sqrt{{\left(x_{PTV}-x_{Iso}\right)}^{2}+{\left(y_{PTV}-y_{Iso}\right)}^{2}+{\left(z_{PTV}-z_{Iso}\right)}^{2}}. \end{array}$$

The graphs showed generally a pattern of decreasing robustness of SI plans (larger *δCI*) to an enormous extent of rotations as the PTV‐isocenter distance increased. Their regression lines indicated a recommended threshold PTV‐isocenter distance of 3.6–3.7 cm for rotational shifts of +2.0${}^{\circ }$ and −2${}^{\circ }$, assuming a maximum allowable *δCI* as 0.2. Owing to this phenomenon, the differences between the two approaches were small when the rotational shifts were small, but the discrepancies increased when the shift was amplified. Gevaert et al.^44^ and Huang et al.^41^ also summarized that a small angular error could result in considerable dosimetric degradation particularly for small targets at a distance from the treatment isocenter using the SI approach. To minimize the risk of compromised percentage target coverage in case of experiencing a large intra‐fractional error in multiple‐target SRT using SI approach, Roper et al. recommended to locate the isocenter closer to the small PTV instead of placing it midway between the PTVs.^28^

The impact of translational and rotational isocenter shifts to the OAR doses was relatively mild with *δD_Max_* of different OARs substantially less than 1 Gy in both MI and SI plans. The largest *δD_Max_* were only revealed as 0.73 Gy and 0.88 Gy of brainstem doses in SI plans after translational shifts of +2 and –2 mm (Table 4), and this result would be expected to have limited clinical significance. However, respective doses to different OARs become noteworthy especially when targets are in proximity. This may result in non‐planned irradiation dose and hence collateral damage to these adjacent structures. For a patient whose brainstem was close to PTV and already received treatment dose close to the dose tolerance of the brainstem, a translational isocenter shift of > 1.5 mm should be avoided especially for the SI approach so as not to further increase the hazard of a potential extra dose of around 1 Gy to the organ bringing its total dose to exceed the tolerance. Similarly, attention should be also put on other OARs that are adjacent to the PTV that the addition of 0.5 Gy resulting from extreme isocenter shift might lead to risk beyond its respective tolerance limit. Additionally, as the shifted‐dose distributions were no longer conformed to the PTVs, it is logical to observe that the impact of isocenter shifts to OARs doses became greater with the increasing magnitude of shifts.

When comparing MI with SI plans, there were not many significant differences in OAR doses caused by isocenter shifts despite higher values of *δD_Max_* resulting in SI plans for the majority of OARs after all types of shifts. Only the differences in the left optic nerve and chiasm showed generally consistent significance after both translational ≥ ± 1 mm and rotational shifts of ≥ ± 1${}^{\circ }$. OARs were usually situated at various locations relative to the PTVs and isocenter. It is believed that the shifts might just contribute to a random effect on the OAR doses,^45^ and there is no definite pattern that any of the treatment approaches would be favored. Nevertheless, an important point to note is that over the highly hypofractionated course of treatment irradiating brain lesions in SRS, the random errors may not be provided with an opportunity to be averaged out; these random errors, therefore, become more significant. The impact of overdose on OARs may be as crucial as errors that underdose a PTV^28^ and hence cannot be underestimated. The steep dose gradient in SRT might imply that more precautions are required to protect the OARs.^27^

All in all, the influence of the intra‐fractional isocenter shifts to both MI and SI plans for SRT of multiple brain metastases could not be viewed as negligible. Problems might arise from loss in PTV coverage and OARs overdose (particularly brainstem) when isocenter shift exceeds translational 1.5 mm or rotational 1.5${}^{\circ }$, with the SI approach being more prone to the impact of shifts when compared with the MI approach. Thus, although the SI approach can offer a shorter treatment time and acceptable dose distributions in SRT for multiple brain metastases, greater effort has to be made to minimize the intra‐fractional errors. This may include the use of image guidance with online position tracking and correction^46^ or increased frequency of monitoring for radiation treatment. On the other hand, further researches on topics such as analysis and estimation of PTV margins to account for the errors and investigation on more frequently fractionated treatment or the adoption of SRT instead of single‐fraction SRS in SI approach to overcome the relatively inferior robustness to shifts based on the rationale that fractionation helps reduce the impact of random errors may pave the way to the future development of the use of SI technique.

## 5 CONCLUSION

In SRT for multiple brain metastases, both MI and SI approaches could produce clinically acceptable dose distributions to PTV and OARs, but the quality in plans using the MI approach was relatively better. In addition, there were dosimetric impacts of isocenter shifts on both approaches, and the effects increased with the increase of the magnitude of the shift. Although similar impacts were shown in plans of both approaches after the application of translational isocenter shifts, SI plans were relatively more vulnerable than MI plans in rotational shifts. In particular, transitional shifts of ≥1.5 mm and rotational shifts ≥1.5${}^{\circ }$ should be avoided so as to maintain acceptable PTV dose coverage and keep the OARs doses within their tolerance. Efforts should be made to reduce the intra‐fractional setup discrepancies.

## AUTHOR CONTRIBUTION

*Study conception and design*: Sylvia S. W. Tsui, Vincent W. C. Wu, Jerry S. C. Cheung. *Data collection*: Sylvia S. W. Tsui. *Analysis and interpretation of results*: Sylvia S. W. Tsui, Vincent W. C. Wu. *Draft manuscript preparation*: Sylvia S. W. Tsui, Vincent W. C. Wu. All authors reviewed the results and approved the final version of the manuscript.

## CONFLICT OF INTEREST

The authors declare no conflicts of interest with respect to the content of this manuscript.

## Data Availability

The data that support the findings of this study are available on request from the corresponding author. The data are not publicly available due to privacy or ethical restrictions.

## Appendix

**Table:** Summary of both multiple‐ and single isocenter treatment plans and the respective CIs obtained

			PTV volumes		Number of beams		Conformity Index (CI) per target	
Patient case	Number of Lesions	Location of brain metastases	Per target	Per patient	Per target (MI plans)	Per patient (SI plans)	MI plans	SI plans
1	2	R parietal	26.39	27.90	7	7	0.85	0.90
		R cerebellum	1.51		7		0.87	0.87
2	2	R parietal	18.06	21.71	6	6	0.88	0.87
		L cerebellum	3.65		7		0.85	0.84
3	2	L parietal	22.72	25.05	7	7	0.89	0.90
		R frontal	2.33		7		0.84	0.82
4	2	Cerebellar vermis	0.9	18.19	6	8	0.84	0.81
		R parietal	17.29		8		0.86	0.86
5	2	L Temporal	17.17	18.30	6	6	0.91	0.87
		L cerebellum	1.13		6		0.86	0.85
6	2	R occipital	2.21	2.62	7	7	0.85	0.83
		R occipital	0.41		6		0.71	0.73
7	2	L frontal	14.15	15.68	7	7	0.86	0.87
		L parietal	1.53		7		0.92	0.85
8	2	L occipital	2.44	2.79	6	6	0.86	0.89
		R occipital	0.35		6		0.92	0.90
9	2	L parietal	14.07	18.60	7	7	0.87	0.92
		R parietal	4.53		7		0.86	0.85
10	2	L occipital	71.01	74.28	6	6	0.89	0.87
		L parietal	3.27		6		0.83	0.84
11	3	R parietal	1.35	4.93	6	6	0.86	0.84
		R parietal	1.91		6		0.84	0.81
		R parietal	1.67		7		0.87	0.84
12	2	R occipital	20.04	22.43	7	7	0.68	0.86
		R frontal	2.39		5		0.83	0.86
13	3	L parietal	1.75	3.13	7	7	0.83	0.74
		R frontal	0.64		7		0.73	0.70
		R parietal	0.74		7		0.75	0.69
14	2	L cerebellum	33.65	55.39	6	6	0.90	0.87
		R cerebellum	21.74		6		0.86	0.87
15	2	R parietal	1.16	3.48	7	7	0.85	0.85
		L cerebellum	2.32		7		0.84	0.83
16	2	R frontal	3.68	4.09	7	7	0.86	0.85
		R frontal	0.41		7		0.72	0.71
17	2	L cerebellum	4.88	8.23	6	6	0.86	0.81
		L cerebellopontine angle	3.35		7		0.87	0.90
18	2	L occipital	0.48	5.00	6	7	0.71	0.72
		R frontal	4.52		7		0.85	0.89
19	2	L temporal	4.87	10.58	7	7	0.86	0.88
		L temporal	5.71		7		0.90	0.87
20	2	L frontal	5.94	6.59	8	8	0.86	0.85
		L frontal	0.65		6		0.87	0.80
21	2	R temporal	1.84	3.51	7	7	0.84	0.82
		R cerebellum	1.67		6		0.85	0.82
22	2	R frontal	1.54	2.19	9	9	0.82	0.85
		R frontal	0.65		4		0.78	0.70

CI = Conformity index

MI = Multiple‐isocenter, SI=Single‐isocenter

R = Right, L = Left
